# The Law of Psychic Phenomena

**Published:** 1894-03

**Authors:** 


*The Law of Psychic Phenomena.* By THOMSON JAY HUDSON. Pp. 409. London: G. P. PUTNAM'S SONS. [1892.]

The main object of *The Law of Psychic Phenomena* is, as its somewhat ambitious title indicates, to provide a working hypothesis in the light of which all classes of mental phenomena shall be interpretable. The main proposition of the hypothesis is that man has two minds—"subjective" and "objective." Mr. Hudson affords us no precise definition of the "subjective" mind; but by means of various scattered remarks we learn that it is an omniscient entity, capable of intuiting fixed laws, independent of brain action, in this life subject to the trammels of sense (represented by the "objective" mind), but rising supreme as the brain becomes weakened and impaired, and finally, with its death, entering on the fulness of immortal life.

It is unfortunate in so good a cause that the theory should include so many mutually exclusive propositions. In the face of recent psycho-physiological researches, it is a little venturesome to maintain that large classes of mental phenomena go on independently of neural processes; but Mr. Hudson does so with an almost wanton lightness. Again, the postulate of intuitive faculties is really not of the axiomatic nature the author would assume.

It would seem that Mr. Hudson has found belief in the immortality of the soul incompatible with that in cerebral concomitants, and that, clinging tenaciously to the former, he has distorted facts and taken up positions which it is impossible to maintain in the light of modern knowledge.

